# Decachloro­hexa-1,5-diene

**DOI:** 10.1107/S1600536812019769

**Published:** 2012-05-12

**Authors:** Dieter Schollmeyer, Heiner Detert

**Affiliations:** a University Mainz, Duesbergweg 10-14, 55099 Mainz, Germany

## Abstract

The title compound, C_6_Cl_10_, cystallizes in a nearly *C*2-symmetrical *gauche* conformation. Both trichloro­vinyl groups are nearly planar [Cl—C—C—Cl torsion angles = −178.47 (12) and −179.93 (11)°] and the lengths of their C—Cl bonds increase from the terminal *trans* and *cis* C—Cl bonds to the inter­nal bonds. The Cl—C—Cl bond angles of the terminal dichloro­methyl­ene units are compressed to 111.75 (11) and 111.40 (11)°.

## Related literature

For the synthesis of perchloro­alkenes, see: Prins (1949 ▶); Roedig *et al.* (1963 ▶). For structures of perchloro­alkenes, see: Herbstein (1979 ▶); Rao & Livingston (1958 ▶); Hopf *et al.* (1991 ▶); Detert *et al.* (2009 ▶). For rearrangements of highly halogenated alkenes, see: Maahs (1963 ▶); Herges *et al.* (2005 ▶). For recent reactions of perchloro­alkenes, see: Schmidt *et al.* (2009 ▶); Rahimi & Schmidt (2010 ▶).
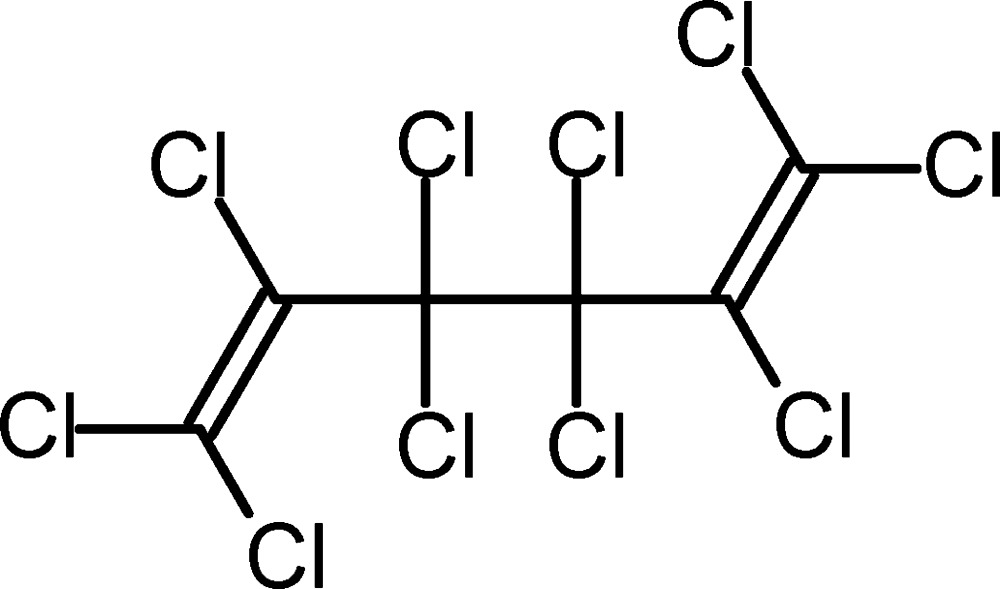


## Experimental

### 

#### Crystal data

C_6_Cl_10_
*M* *_r_* = 426.56
Monoclinic, 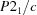

*a* = 12.8936 (5) Å
*b* = 6.7051 (2) Å
*c* = 15.3753 (5) Å
β = 93.858 (3)°
*V* = 1326.23 (8) Å^3^
*Z* = 4
Mo *K*α radiation
μ = 2.07 mm^−1^
*T* = 193 K
0.15 × 0.15 × 0.15 mm

#### Data collection

Stoe IPDS 2T diffractometer
Absorption correction: multi-scan (*PLATON*; Spek, 2009 ▶) *T*_min_ = 0.747, *T*_max_ = 0.747
18142 measured reflections
3181 independent reflections
2991 reflections with *I* > 2σ(*I*)
*R*_int_ = 0.044

#### Refinement

*R*[*F* ^2^ > 2σ(*F* ^2^)] = 0.030
*wR*(*F* ^2^) = 0.075
*S* = 1.06
3181 reflections
145 parameters
Δρ_max_ = 0.94 e Å^−3^
Δρ_min_ = −0.44 e Å^−3^

### 

Data collection: *X-AREA* (Stoe & Cie, 2011 ▶); cell refinement: *X-AREA*; data reduction: *X-RED* (Stoe & Cie, 2011 ▶); program(s) used to solve structure: *SIR97* (Altomare *et al.* 1999 ▶); program(s) used to refine structure: *SHELXL97* (Sheldrick, 2008 ▶); molecular graphics: *PLATON* (Spek, 2009 ▶); software used to prepare material for publication: *PLATON*.

## Supplementary Material

Crystal structure: contains datablock(s) I, global. DOI: 10.1107/S1600536812019769/bt5907sup1.cif

Structure factors: contains datablock(s) I. DOI: 10.1107/S1600536812019769/bt5907Isup2.hkl

Supplementary material file. DOI: 10.1107/S1600536812019769/bt5907Isup3.cml

Additional supplementary materials:  crystallographic information; 3D view; checkCIF report

## Figures and Tables

**Fig. 1.**
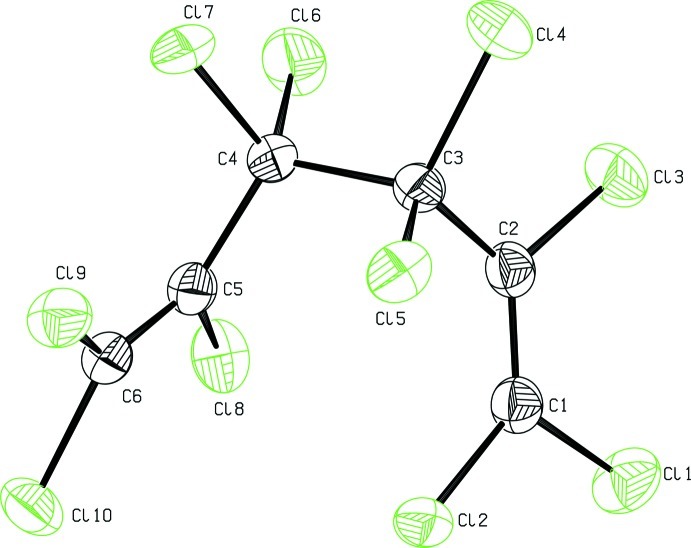
View of compound I. Displacement ellipsoids are drawn at the 50% probability level.

## supplementary crystallographic information

### Comment

In the monoclinic crystal, decachlorohexadiene adopts a *gauche* conformation [C2—C3—C4—C5: 60.4 (2)°] with a non-perfect C2-symmetry. With torsion angles of -178.47 (12)° (Cl2—C1—C2—Cl3) and -179.93 (11)° (Cl8—C5—C6—Cl9) both trichorovinyl groups are nearly planar. The C—Cl bonds of these units are significantly different. The bond lengths C2—Cl3 [1.736 (2) Å] and C5—Cl8 [1.737 (2) Å] are sligthly longer than the corresponding bonds (1.731 Å) in *trans*-octachloro-1,3,5-hexatriene (Detert *et al.*, 2009). The bonds to the *cis*-chlorine atoms are shorter: C1—Cl1: 1.721 (2) Å and C6—Cl10: 1.718 (2) Å and those to the *trans*-chlorine atoms are reduced to C1—Cl2: 1.700 (2) Å and C6—Cl9: 1.702 (2). The same bond length variations, but to a lower degree, were found in the triene. With 111.40 (11)° and 111.74 (11)° the bond angles of the terminal dichloromethylene units are smaller than in the reference compound (115.5°).

### Experimental

1,5-Decachlorohexadiene: The diene was prepared from hexachloropropene with cuprous chloride as the coupling agent according the procedure given by Prins. (Prins, 1949) Single crystals were obtained by slow evaporation of a solution of perchlorohexadiene in dichloromethane/methanol.

### Refinement

All atoms were refined with anisotropic displacement parameters.

### Crystal data

**Table:**

C_6_Cl_10_	*F*(000) = 824
*M**_r_* = 426.56	*D*_x_ = 2.136 Mg m^−^^3^
Monoclinic, *P*2_1_/*c*	Mo *K*α radiation, λ = 0.71069 Å
Hall symbol: -P 2ybc	Cell parameters from 28472 reflections
*a* = 12.8936 (5) Å	θ = 2.6–32.4°
*b* = 6.7051 (2) Å	µ = 2.07 mm^−^^1^
*c* = 15.3753 (5) Å	*T* = 193 K
β = 93.858 (3)°	Block, colourless
*V* = 1326.23 (8) Å^3^	0.15 × 0.15 × 0.15 mm
*Z* = 4	

### Data collection

**Table:**

Stoe IPDS 2T diffractometer	3181 independent reflections
Radiation source: sealed X-ray tube, 12 x 0.4 mm long-fine focus	2991 reflections with *I* > 2σ(*I*)
Graphite monochromator	*R*_int_ = 0.044
Detector resolution: 6.67 pixels mm^-1^	θ_max_ = 28.0°, θ_min_ = 3.0°
rotation method scans	*h* = −17→17
Absorption correction: multi-scan (*PLATON*; Spek, 2009)	*k* = −8→8
*T*_min_ = 0.747, *T*_max_ = 0.747	*l* = −20→19
18142 measured reflections	

### Refinement

**Table:**

Refinement on *F*^2^	0 restraints
Least-squares matrix: full	Primary atom site location: structure-invariant direct methods
*R*[*F*^2^ > 2σ(*F*^2^)] = 0.030	Secondary atom site location: difference Fourier map
*wR*(*F*^2^) = 0.075	*w* = 1/[σ^2^(*F*_o_^2^) + (0.0314*P*)^2^ + 1.2539*P*] where *P* = (*F*_o_^2^ + 2*F*_c_^2^)/3
*S* = 1.06	(Δ/σ)_max_ < 0.001
3181 reflections	Δρ_max_ = 0.94 e Å^−^^3^
145 parameters	Δρ_min_ = −0.44 e Å^−^^3^

### Special details

**Table:**

Experimental. ^13^C-NMR (75 MHz, CDCl_3_): δ = 94.5 (C-3,4), 128.1 (C-1,6), 131.2 (C-2,5)MS (FD): 426 (100%, Cl10 pattern) [*M*]^+.^C_6_Cl_10_ (426.596): calcd. C 16.89%; found C 17.06%.
Geometry. All e.s.d.'s (except the e.s.d. in the dihedral angle between two l.s. planes) are estimated using the full covariance matrix. The cell e.s.d.'s are taken into account individually in the estimation of e.s.d.'s in distances, angles and torsion angles; correlations between e.s.d.'s in cell parameters are only used when they are defined by crystal symmetry. An approximate (isotropic) treatment of cell e.s.d.'s is used for estimating e.s.d.'s involving l.s. planes.
Refinement. Refinement of *F*^2^ against ALL reflections. The weighted *R*-factor *wR* and goodness of fit *S* are based on *F*^2^, conventional *R*-factors *R* are based on *F*, with *F* set to zero for negative *F*^2^. The threshold expression of *F*^2^ > σ(*F*^2^) is used only for calculating *R*-factors(gt) *etc*. and is not relevant to the choice of reflections for refinement. *R*-factors based on *F*^2^ are statistically about twice as large as those based on *F*, and *R*- factors based on ALL data will be even larger.

### Fractional atomic coordinates and isotropic or equivalent isotropic displacement parameters (Å^2^)

**Table:**

	*x*	*y*	*z*	*U*_iso_*/*U*_eq_	
Cl1	0.07308 (4)	0.63553 (10)	0.19647 (4)	0.04455 (14)	
Cl2	0.28741 (4)	0.54432 (8)	0.22272 (3)	0.03407 (12)	
Cl3	0.03427 (4)	0.60643 (9)	0.38657 (4)	0.04477 (14)	
Cl4	0.20468 (5)	0.63372 (9)	0.52864 (4)	0.04909 (16)	
Cl5	0.36486 (4)	0.62962 (8)	0.41150 (3)	0.03657 (12)	
Cl6	0.13705 (5)	0.18663 (9)	0.47450 (4)	0.04778 (15)	
Cl7	0.34462 (5)	0.25344 (10)	0.54017 (3)	0.04564 (15)	
Cl8	0.19658 (4)	0.09671 (7)	0.29308 (4)	0.03836 (13)	
Cl9	0.50500 (4)	0.20720 (9)	0.40404 (3)	0.03898 (13)	
Cl10	0.41762 (4)	0.02630 (8)	0.25017 (4)	0.04048 (13)	
C1	0.17431 (15)	0.5804 (3)	0.27135 (13)	0.0302 (4)	
C2	0.16004 (15)	0.5674 (3)	0.35642 (13)	0.0290 (4)	
C3	0.24229 (15)	0.5212 (3)	0.43068 (12)	0.0287 (4)	
C4	0.25883 (15)	0.2894 (3)	0.44660 (12)	0.0294 (4)	
C5	0.29541 (15)	0.1796 (3)	0.36660 (12)	0.0281 (4)	
C6	0.39289 (15)	0.1447 (3)	0.34565 (12)	0.0289 (4)	

### Atomic displacement parameters (Å^2^)

**Table:**

	*U*^11^	*U*^22^	*U*^33^	*U*^12^	*U*^13^	*U*^23^
Cl1	0.0363 (3)	0.0573 (3)	0.0386 (3)	0.0024 (2)	−0.0082 (2)	0.0092 (2)
Cl2	0.0338 (2)	0.0402 (3)	0.0291 (2)	0.00294 (19)	0.00900 (17)	0.00380 (18)
Cl3	0.0355 (3)	0.0501 (3)	0.0508 (3)	0.0077 (2)	0.0182 (2)	0.0015 (2)
Cl4	0.0693 (4)	0.0454 (3)	0.0335 (3)	0.0115 (3)	0.0101 (2)	−0.0131 (2)
Cl5	0.0408 (3)	0.0329 (2)	0.0352 (2)	−0.00885 (19)	−0.00350 (19)	0.00085 (19)
Cl6	0.0460 (3)	0.0377 (3)	0.0628 (4)	−0.0004 (2)	0.0267 (3)	0.0081 (3)
Cl7	0.0566 (3)	0.0565 (3)	0.0241 (2)	0.0145 (3)	0.0046 (2)	0.0129 (2)
Cl8	0.0339 (2)	0.0287 (2)	0.0518 (3)	−0.00371 (18)	−0.0026 (2)	−0.0083 (2)
Cl9	0.0299 (2)	0.0521 (3)	0.0345 (2)	0.0026 (2)	−0.00126 (18)	0.0047 (2)
Cl10	0.0467 (3)	0.0384 (3)	0.0377 (3)	0.0036 (2)	0.0133 (2)	−0.0090 (2)
C1	0.0304 (9)	0.0270 (9)	0.0333 (9)	0.0010 (7)	0.0030 (7)	0.0006 (7)
C2	0.0299 (9)	0.0239 (8)	0.0335 (9)	0.0010 (7)	0.0051 (7)	−0.0005 (7)
C3	0.0372 (9)	0.0256 (9)	0.0235 (8)	0.0017 (7)	0.0031 (7)	−0.0025 (7)
C4	0.0339 (9)	0.0281 (9)	0.0271 (8)	0.0043 (7)	0.0079 (7)	0.0055 (7)
C5	0.0347 (9)	0.0214 (8)	0.0284 (8)	0.0004 (7)	0.0037 (7)	0.0014 (7)
C6	0.0328 (9)	0.0260 (9)	0.0280 (8)	0.0015 (7)	0.0030 (7)	0.0026 (7)

### Geometric parameters (Å, º)

**Table:**

Cl1—C1	1.721 (2)	Cl9—C6	1.702 (2)
Cl2—C1	1.700 (2)	Cl10—C6	1.718 (2)
Cl3—C2	1.736 (2)	C1—C2	1.336 (3)
Cl4—C3	1.7806 (19)	C2—C3	1.536 (3)
Cl5—C3	1.782 (2)	C3—C4	1.586 (3)
Cl6—C4	1.793 (2)	C4—C5	1.535 (3)
Cl7—C4	1.771 (2)	C5—C6	1.339 (3)
Cl8—C5	1.737 (2)		
			
C2—C1—Cl2	126.87 (16)	C5—C4—C3	113.08 (15)
C2—C1—Cl1	121.38 (16)	C5—C4—Cl7	112.13 (13)
Cl2—C1—Cl1	111.75 (11)	C3—C4—Cl7	109.21 (14)
C1—C2—C3	127.38 (18)	C5—C4—Cl6	109.18 (14)
C1—C2—Cl3	116.37 (15)	C3—C4—Cl6	107.63 (13)
C3—C2—Cl3	116.25 (14)	Cl7—C4—Cl6	105.21 (10)
C2—C3—C4	113.02 (15)	C6—C5—C4	128.36 (18)
C2—C3—Cl4	109.25 (13)	C6—C5—Cl8	116.56 (15)
C4—C3—Cl4	109.03 (13)	C4—C5—Cl8	115.05 (14)
C2—C3—Cl5	111.74 (13)	C5—C6—Cl9	127.43 (16)
C4—C3—Cl5	108.28 (13)	C5—C6—Cl10	121.17 (16)
Cl4—C3—Cl5	105.21 (10)	Cl9—C6—Cl10	111.40 (11)
			
Cl2—C1—C2—C3	1.4 (3)	Cl5—C3—C4—Cl7	61.66 (14)
Cl1—C1—C2—C3	−179.52 (15)	C2—C3—C4—Cl6	−60.28 (18)
Cl2—C1—C2—Cl3	−178.47 (12)	Cl4—C3—C4—Cl6	61.42 (15)
Cl1—C1—C2—Cl3	0.7 (2)	Cl5—C3—C4—Cl6	175.38 (9)
C1—C2—C3—C4	−86.2 (2)	C3—C4—C5—C6	90.7 (2)
Cl3—C2—C3—C4	93.61 (17)	Cl7—C4—C5—C6	−33.3 (3)
C1—C2—C3—Cl4	152.21 (18)	Cl6—C4—C5—C6	−149.47 (18)
Cl3—C2—C3—Cl4	−27.96 (18)	C3—C4—C5—Cl8	−87.37 (18)
C1—C2—C3—Cl5	36.2 (3)	Cl7—C4—C5—Cl8	148.61 (11)
Cl3—C2—C3—Cl5	−143.95 (11)	Cl6—C4—C5—Cl8	32.41 (17)
C2—C3—C4—C5	60.4 (2)	C4—C5—C6—Cl9	2.0 (3)
Cl4—C3—C4—C5	−177.90 (14)	Cl8—C5—C6—Cl9	−179.93 (11)
Cl5—C3—C4—C5	−63.94 (18)	C4—C5—C6—Cl10	−177.43 (15)
C2—C3—C4—Cl7	−174.00 (13)	Cl8—C5—C6—Cl10	0.7 (2)
Cl4—C3—C4—Cl7	−52.30 (16)		
